# Ileal obstruction caused by an intact “Shine Muscat” grape: a case report

**DOI:** 10.3389/fmed.2025.1546570

**Published:** 2025-05-15

**Authors:** Kuanyong Yu, Wenjun Feng, Guanghui Qiang, Chuanyang Cao, Liyang Liu, Zhenling Ji

**Affiliations:** Department of General Surgery, Nanjing Jiangbei Hospital, Nanjing, China

**Keywords:** small-bowel obstruction, foreign body, “Shine Muscat” grape, elderly, computed tomography (CT) scan, case report

## Abstract

**Introduction:**

This case report presents a rare instance of small-bowel obstruction (SBO) caused by an intact “Shine Muscat” grape, highlighting the importance of considering unusual foreign bodies in the differential diagnosis of SBO, especially in the elderly.

**Case description:**

An 87-year-old edentulous female presented with a 2-day history of abdominal pain, bloating, vomiting, and constipation. Abdominal computed tomography (CT) scan revealed SBO with a foreign body in the ileum. The patient confessed swallowing an intact grape 2 days prior to admission. Surgery was performed, and an intact green grape was extracted from the dilated ileum, relieving the obstruction. This case highlights the rare case of SBO caused by an intact grape and underscores the importance of a detailed dietary history in the diagnosis of SBO. The use of CT scanning was instrumental in identifying the grape as the cause of obstruction. Early surgical intervention led to a successful outcome, emphasizing the need for prompt action in managing such cases.

## Introduction

Small bowel obstruction (SBO) is a common surgical emergency associated with significant morbidity and mortality. It accounts for up to 20% of general surgical admissions and carries a mortality risk of approximately 10% within 30 days of diagnosis (1). The etiology of SBO is diverse, with adhesions, hernias, and malignancy being the most common causes (2). However, foreign bodies, particularly food items, can also lead to SBO, albeit less commonly (3). The ingestion of intact foreign objects, such as fruits, can result in life-threatening bowel obstruction, especially in the older adult population with compromised gastrointestinal motility (4). Here, we present a unique case of SBO caused by an intact “Shine Muscat” grape, with the aim of providing guidance for clinical physicians, as such cases are rarely reported in the literature.

## Case report

**Figure 1:**
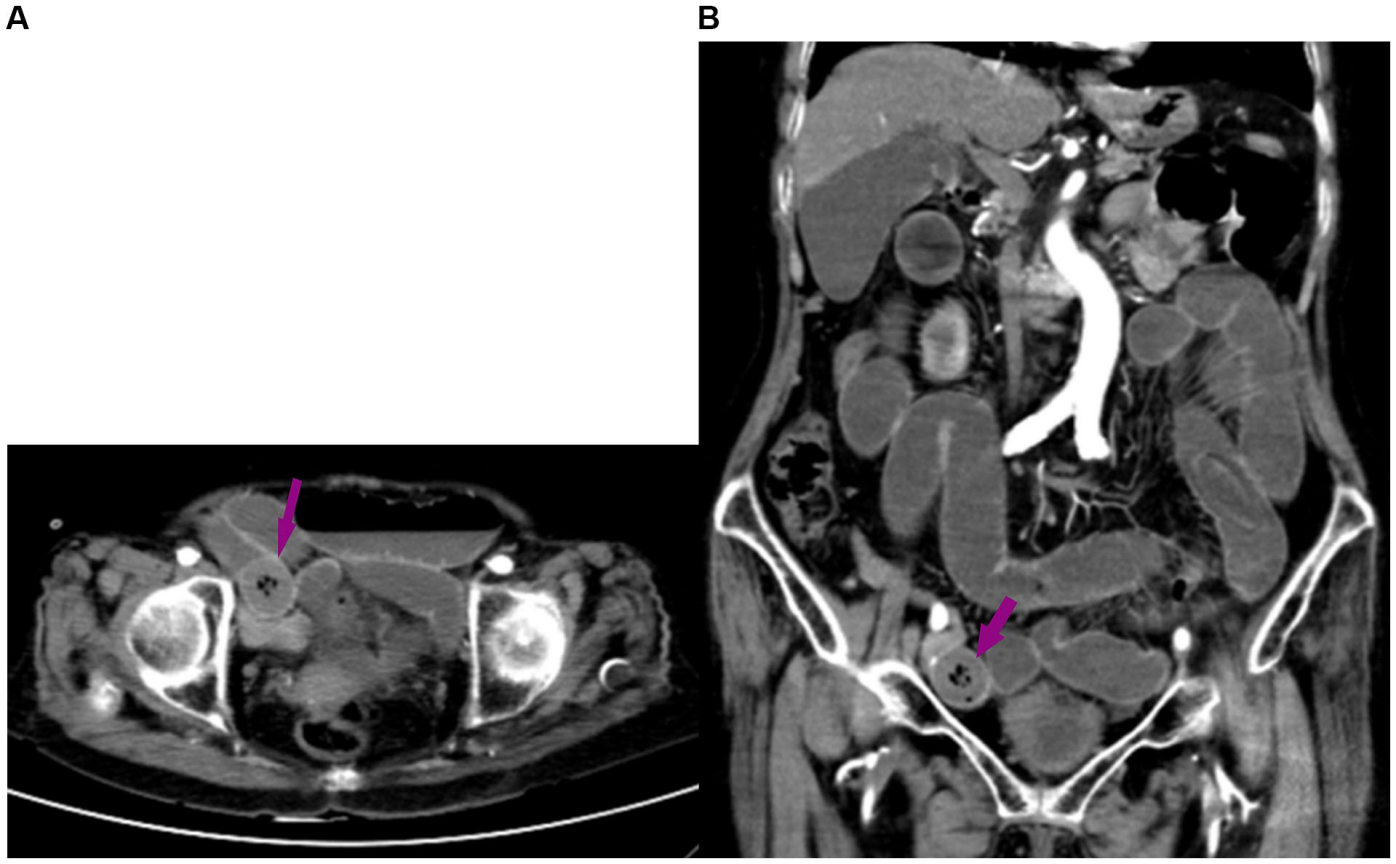
**(A)** Abdominal computed tomography (CT) scan showing small bowel obstruction, with a foreign body (arrow) in the abdomen. **(B)** The abdominal contrast-enhanced CT scan revealed a round, isodense lesion in the small intestine of the right lower abdomen, suggestive of a foreign body, with secondary obstruction of the proximal small intestine.

An 87-year-old female presented with abdominal pain, bloating, vomiting, and constipation that lasted for 2 days. She had no history of abdominal surgery. The abdominal plain computed tomography (CT) revealed SBO with a mass in the abdomen (Figure 1A). The patient was transferred from the emergency department to the department of general surgery. Then, a contrast-enhanced CT scan was performed to rule out vascular issues. The abdominal contrast-enhanced CT scan showed a round, isodense lesion in the right lower abdomen, consistent with a foreign body, causing secondary obstruction of the proximal small intestine (Figure 1B). Upon further questioning, the patient admitted to regularly binging on grapes and confessed that she had accidentally swallowed one intact grape 2 days before. The patient had been bedridden since undergoing intramedullary nailing for a left intertrochanteric fracture 2 years ago. The patient also underwent percutaneous vertebroplasty for a lumbar vertebral compression fracture 5 months ago.

The physical examination revealed edentulousness, abdominal distention, and tenderness. The laboratory results showed an elevated neutrophil ratio of 80.5% (normal range, 40%–75%) and an increased hypersensitive C-reactive protein (CRP) level of 57.1 mg/L (normal range, <10 mg/L). The serum albumin level was reduced to 27.3 g/L (normal range, 40–45 g/L), and the serum calcium concentration was lowered to 1.73 mmol/L (normal range, 2.11–2.52 mmol/L). Given the patient’s history of ingesting a whole grape, we primarily diagnosed SBO, likely caused by the grape. The possibility of a neoplasm was also considered. Despite conservative treatments, such as gastrointestinal decompression, antibiotic therapy, fluid resuscitation, and fasting, the patient’s abdominal pain and distension worsened over 8 h. We then discussed the condition with the family, outlining the risks of continued conservative management, such as bowel perforation, peritonitis, ischemic bowel, surgical delay, symptom progression, and psychological stress. We also explained the risks and benefits of laparoscopy. The family requested to proceed with laparoscopic surgery.

**Figure 2:**
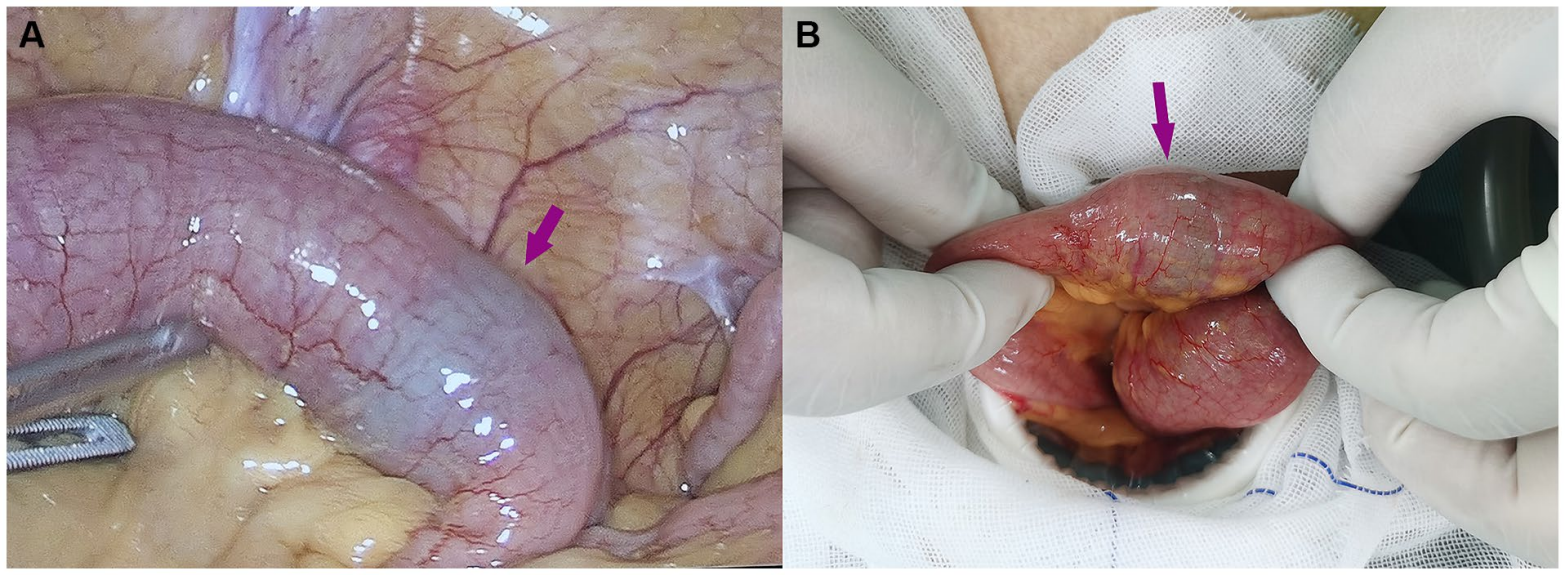
**(A)** A laparoscopic examination was performed, and a green foreign body was found embedded in the lumen of the dilated ileum approximately 20 cm away from the ileocecal region. **(B)** The obstructed segment of the small intestine.

**Figure 3:**
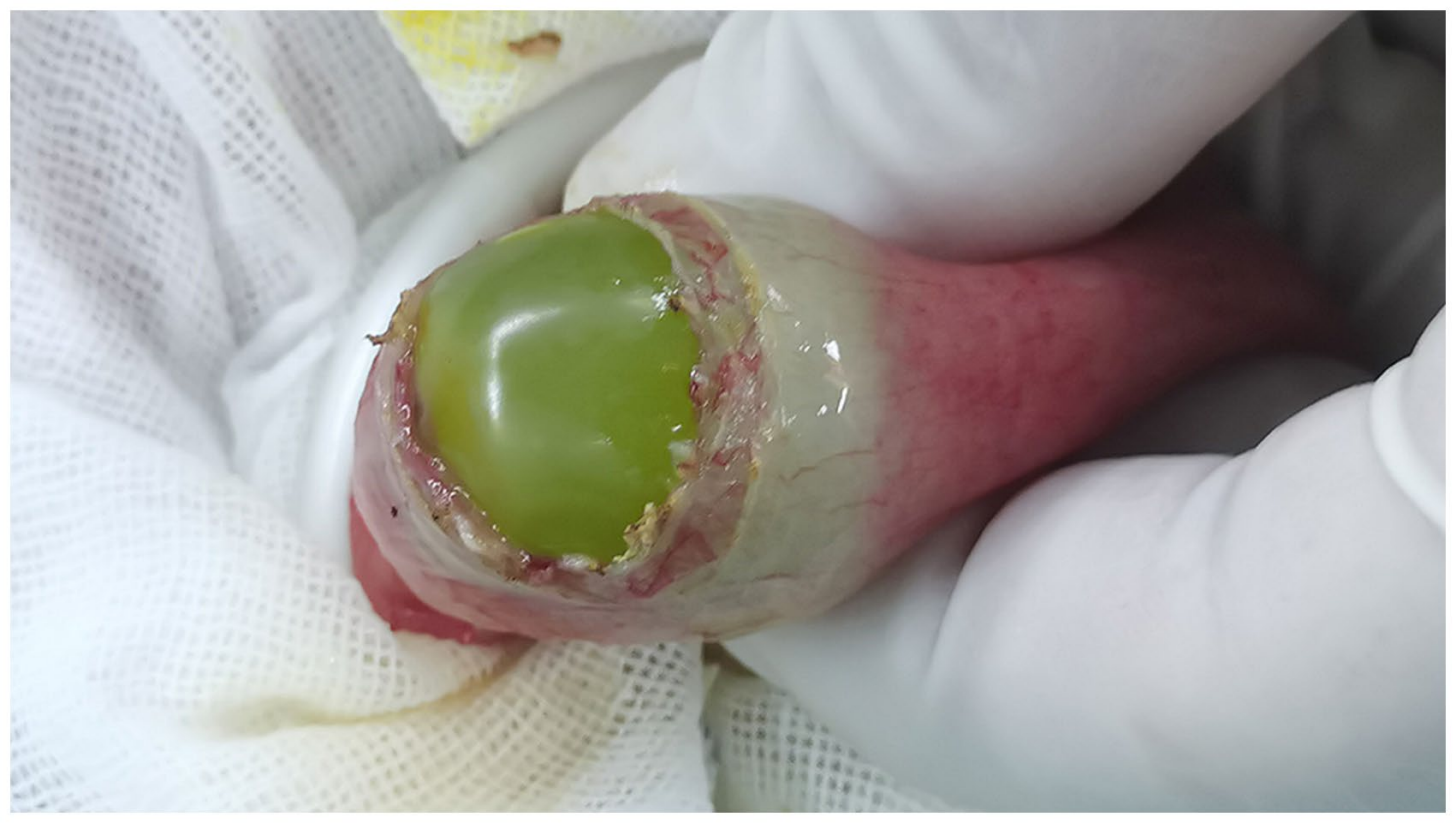
A 2-cm transverse incision along the antimesenteric border of the bowel was made, and an intact fresh grape was extracted.

A laparoscopic examination was performed, and a green foreign body was found embedded in the lumen of the dilated ileum approximately 20 cm away from the ileocecal region (Figure 2A). An auxiliary 4-cm McBurney incision was made to pull out the obstructed segment loop of the ileum (Figure 2B). The grape was pushed forward into the distal, normal ileum. The area around the incision was protected with gauze. Then, a 2-cm transverse incision was made along the antimesenteric border of the bowel, and an intact fresh grape was extracted (Figure 3). The enterotomy was closed with 4–0 Vicryl sutures. A drainage tube was placed in the abdomen, and the incision was closed routinely.

**Figure 4:**
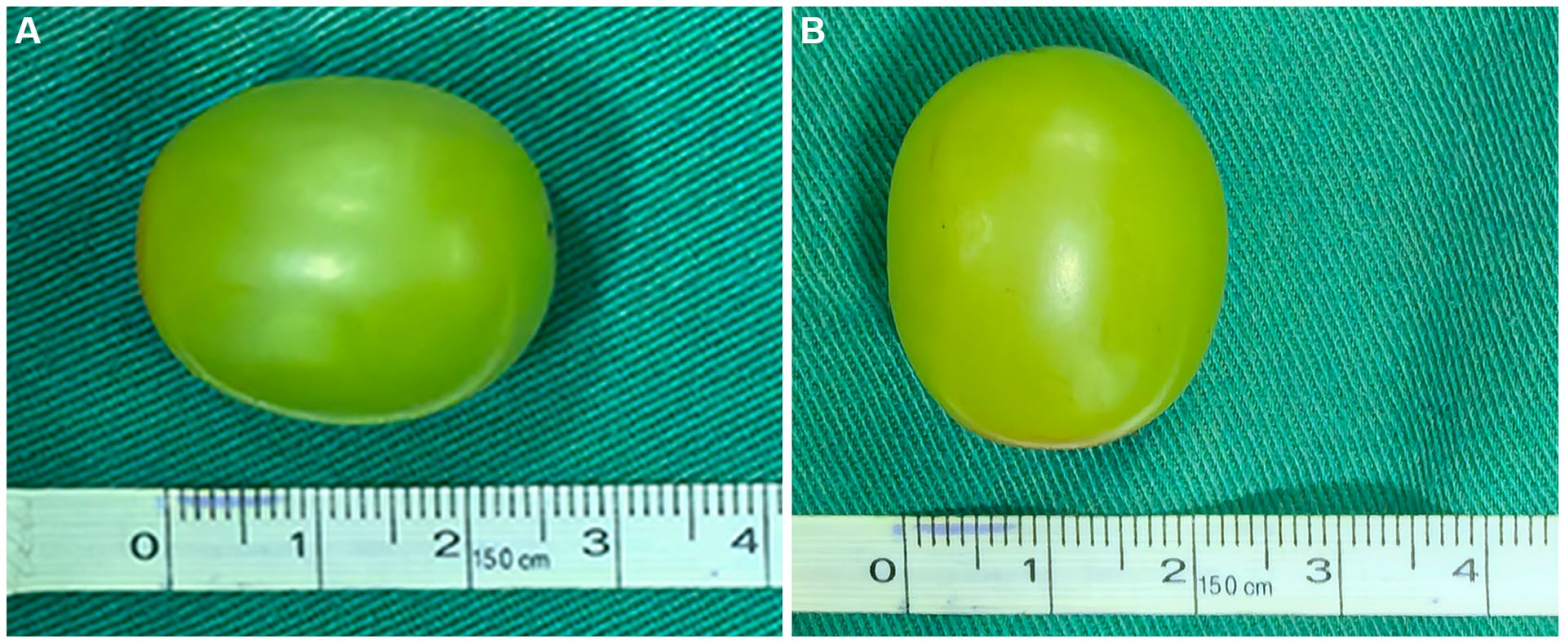
The grape was approximately 2.6 cm in length **(A)** and 2 cm in width **(B)**.

The grape was measured to be approximately 2.6 cm × 2.0 cm in size (Figures 4A,B). The patient recovered uneventfully and was discharged on the seventh postoperative day. After discharge, we followed-up with the patient by phone for 5 months. During this time, the patient experienced no recurrent obstructions or other symptoms, showing good overall recovery.

## Discussion

SBO is a surgical emergency. Adhesions, hernias, and tumors are the most common causes (1), while foreign bodies are a frequent cause in children, older adults with dental prosthesis, alcoholics, and psychiatric patients. Phytobezoars, rare causes of SBO, are undigested masses of cellulose- or tannin-rich vegetables or fruits (5). In this case, the patient’s SBO was caused by swallowing a whole fresh grape. Such a situation is extremely rare, and there have been only three documented cases in the literature (6–8). Our patient was an edentulous older individual, and the obstruction was located in the terminal ileum, which is consistent with previous reports (6, 7). This is likely because the terminal ileum and ileocecal valve, with their narrow diameter and weak peristalsis, are the gastrointestinal regions most susceptible to obstruction (9). Our case contributes to the very limited literature on SBO caused by an entire piece of fruit. However, our report is limited by its single-case nature, which restricts the generalizability of our findings.

Consistent with the report by Gu et al. (8), the grape in this case was a Shine Muscat, characterized by its yellow-green, thin skin and hard, crisp flesh (10). Grape skins are rich in a variety of nutrients, such as anthocyanins, resveratrol, cellulose, pectin, and vitamins. We speculate that certain components of the grape skin, such as abundant cellulose, are indigestible, potentially leading to obstruction. Similar to SBO caused by other factors, this patient’s main symptoms were abdominal pain, bloating, vomiting, and constipation. CT imaging offers a superior approach for diagnosing intestinal foreign bodies, as it can avoid the disturbance caused by intestinal gases and feces (11). In our case, an abdominal two-dimensional CT scan was performed, revealing a round, isodense lesion in the small intestine of the right lower abdomen, suggestive of a foreign body, with secondary obstruction of the proximal small intestine. Detailed medical history, especially information on food consumption, is important for suspecting a food bolus-induced bowel obstruction. Fruits such as persimmon are a common cause, but grapes can also be a potential cause in certain patients, which is an important consideration.

For patients with SBO, the World Society of Emergency Surgery (WSES) recommends attempting non-surgical treatment if there is no evidence of peritonitis, strangulation, or bowel ischemia. Although there is no definitive evidence regarding the optimal duration of non-surgical treatment, most authors and expert panels consider a 72-h period to be safe and appropriate (12). However, delaying surgery for more than 2 days in older adult patients with SBO increases the risk of mortality (13). Given the patient’s advanced age and worsening abdominal pain despite conservative treatment, continuing with the conservative treatment carried a significant risk of bowel perforation and peritonitis. After a discussion with the patient and her family, the decision was made to proceed with surgery.

There are four main treatment options for intestinal obstruction caused by bezoars, namely chemical dissolution, endoscopic fragmentation and removal, laparoscopic surgery, and open surgery. The choice of treatment depends on the bezoar’s size, material, location, and associated complications (5). There have been reports of managing SBO caused by the ingestion of foreign bodies through upper or lower gastrointestinal endoscopy. However, at present, the available guidelines on ingested foreign bodies mainly focus on endoscopic management of objects located in the upper gastrointestinal tract (14). In this case, the obstruction was caused by a fresh whole grape, and therefore, we opted for a laparoscopic examination.

Laparoscopic surgery is not a contraindication in patients with ileus. However, the insertion of trocars should be carried out carefully to avoid injury to the viscera. It has been reported that patients with intestinal wall edema have an increased risk of anastomotic leakage (15). In this case, an enterotomy was performed on the normal small intestine, distal to the obstruction site, which helped reduce the risk of anastomotic leakage. Gu et al. (8) reported a case of intestinal obstruction caused by grape ingestion in a 7-month-old infant. The surgical team used minimally invasive laparoscopic techniques to crush the grape and move it to the colon. The child recovered well after the surgery, and the grape fragments were passed naturally. In our case, we removed the grape from the gastrointestinal tract, in accordance with the surgical methods described by Cox (6) and Trotta (7), considering that we could not determine whether its presence in the colon would cause another obstruction. We had thorough discussions with the patient and her family before the surgery, and they requested the removal of the grape. We speculate that pushing the grape into the colon and crushing it could be a viable option, but this approach requires further research to confirm its efficacy and safety.

This report emphasizes that in older adult patients, the combination of decreased gastrointestinal motility and dental issues can lead to rare instances of SBO caused by swallowing whole foods such as grapes. This case highlights the importance of thorough medical history taking—particularly regarding dietary intake—and the crucial role of two-dimensional CT scans in accurately diagnosing grape-induced bowel obstruction. Early laparoscopic exploration is often necessary for older adult patients to avoid the high mortality risk associated with delayed surgery. Furthermore, this case serves as a reminder for clinicians to consider non-traditional causes, such as food-related obstructions, when managing SBO and to engage in comprehensive communication with patients and their families to determine the best treatment strategy. Ultimately, we successfully removed the obstructing grape through surgery, and the patient recovered well, confirming that surgical intervention can be an effective treatment option for such obstructions in certain cases.

## Patient perspective

As an 87-year-old woman, I went through an extraordinary medical experience. Two days before, I experienced SBO after accidentally swallowing an intact “Shine Muscat” grape, which caused me great distress and discomfort. I experienced abdominal pain, bloating, vomiting, and constipation for 2 days. After an abdominal CT scan identified the cause of my obstruction, the medical team decided to proceed with surgery. During the procedure, they carefully extracted the intact grape from my dilated ileum, which brought me immense relief. I recovered well after the surgery, without any complications, and I was discharged on the seventh postoperative day.

This treatment experience has made me deeply appreciate the professionalism and care of the medical team. Not only did they promptly and accurately diagnose my condition, but they also took effective surgical measures that led to my swift recovery. I am sincerely grateful for the exceptional medical skills and patient care provided by the doctors and nurses. This experience also serves as a reminder that even seemingly harmless daily habits can lead to unexpected health risks. I will be more mindful of my dietary habits to avoid such situations in the future.

## Data Availability

The original contributions presented in the study are included in the article/supplementary material, further inquiries can be directed to the corresponding author.

## References

[1] Lee MJ Sayers AE Drake TM Marriott PJ Anderson ID Bach SP. National prospective cohort study of the burden of acute small bowel obstruction. BJS Open. (2019) 3:354–66. doi: 10.1002/bjs5.50136, PMID: 31183452 PMC6551410

[2] Olausson M Aerenlund MP Azzam M Bjerke T Burcharth JFH Dibbern CB. Management and short-term outcomes of patients with small bowel obstruction in Denmark: a multicentre prospective cohort study. Eur J Trauma Emerg Surg. (2023) 49:1121–30. doi: 10.1007/s00068-022-02171-y, PMID: 36357790 PMC9648885

[3] Behman R Nathens AB Mason S Byrne JP Hong NL Pechlivanoglou P. Association of surgical intervention for adhesive small-bowel obstruction with the risk of recurrence. JAMA Surg. (2019) 154:413–20. doi: 10.1001/jamasurg.2018.5248, PMID: 30698610 PMC6537786

[4] Firth M Prather CM. Gastrointestinal motility problems in the elderly patient. Gastroenterology. (2002) 122:1688–700. doi: 10.1053/gast.2002.33566 12016432

[5] Albostani A Kfelati F Alsaadi W Faraman RA Farman A. Small bowel obstruction due to a meat bolus bezoar: the second case report in literature. Ann Med Surg (Lond). (2024) 86:1139–43. doi: 10.1097/MS9.0000000000001633, PMID: 38333246 PMC10849409

[6] Cox J Grigg M. Small bowel obstruction by an intact grape. J Am Geriatr Soc. (1986) 34:550. doi: 10.1111/j.1532-5415.1986.tb04250.x, PMID: 3722673

[7] Trotta M Cesaretti M Conzi R Derchi LE Borgonovo G. Elderly male with mesogastric pain. Small bowel obstruction caused by an intact fresh grape. Ann Emerg Med. (2011) 58:e1–2. doi: 10.1016/j.annemergmed.2011.04.031, PMID: 21943590

[8] Gu C Zhang Y Jiang G Hu X. Diagnosis of small bowel obstruction due to Shine-Muscat grape ingestion: case report. Front Pediatr. (2024) 12:1503456. doi: 10.3389/fped.2024.1503456, PMID: 39748813 PMC11693656

[9] Reisner RM Cohen JR. Gallstone ileus: a review of 1001 reported cases. Am Surg. (1994) 60:441–6. PMID: 8198337

[10] Wei X Xie F Wang K Song J Bai Y. A study on Shine-Muscat grape detection at maturity based on deep learning. Sci Rep. (2023) 13:4587. doi: 10.1038/s41598-023-31608-6, PMID: 36941309 PMC10027863

[11] Lai JQ Xu YN. Small bowel obstruction caused by a rare foreign body: a case report and literature review. Curr Med Imaging. (2024) 20:e15734056339263. doi: 10.2174/0115734056339263240826103827, PMID: 39219118

[12] Ten Broek RPG Krielen P Di Saverio S Coccolini F Biffl WL Ansaloni L. Bologna guidelines for diagnosis and management of adhesive small bowel obstruction (ASBO): 2017 update of the evidence-based guidelines from the world society of emergency surgery ASBO working group. World J Emerg Surg. (2018) 13:24. doi: 10.1186/s13017-018-0185-2, PMID: 29946347 PMC6006983

[13] Li R Quintana MT Lee J Sarani B Kartiko S. Timing to surgery in elderly patients with small bowel obstruction: an insight on frailty. J Trauma Acute Care Surg. (2024) 97:623–30. doi: 10.1097/TA.0000000000004410, PMID: 38787701

[14] Taylor JE Campbell M Daley B. The management of small bowel obstruction caused by ingested gastrostomy tube. Am Surg. (2019) 85:e372–3. doi: 10.1177/000313481908500802, PMID: 31560316

[15] Spinelli A Anania G Arezzo A Berti S Bianco F Bianchi PP. Italian multi-society modified Delphi consensus on the definition and management of anastomotic leakage in colorectal surgery. Updat Surg. (2020) 72:781–92. doi: 10.1007/s13304-020-00837-z, PMID: 32613380

